# Mr. Fox, on Stopping Decayed Teeth

**Published:** 1807-12

**Authors:** Joseph Fox

**Affiliations:** Dover


### *To the Editors of the Medical and Physical Journal.*

Gentlemen,

In the Medical and Physical Journal for September last, p. 210, Mr. Wawn, of Newcastle, has been so obliging as to make some remarks upon "the new method of stopping teeth," noticed in my last publication upon the Diseases of the Teeth; and as the objections which that Gentleman offers to this practice are so very proper, I shall take the liberty to state the result of the success, which I have known to attend the method proposed.

Mr. Pepys was the gentleman from whom I first received the idea of stopping the hollow cavities of teeth, by using the fusible metal; and, at the same time, he shewed me two or three teeth in his own mouth, which, for a considerable time, he had preserved in that manner. Since then, I know that he has performed this operation for several of his friends; and Mr. R. Phillips, of the Poultry, an accurate chemist, had a tooth preserved in this manner for a long time. I must observe, with respect to myself, that excepting (ex capite) in order to shew the mode of performing this operation, in illustration of the subject, in the course of my Lectures at Guy's Hospital *, I have never practised it.

* We are happy to be able to announce, that it is Mr. Fox's intention to deliver his Lectures in the course of the spring; but that, for the perfect re-establishment of his health, he intends to pass the winter at Dover. Ed.

The

The cases in which the fusible metal can be used are so few; and as it is not easy to persuade persons, not acquainted with chemical processes, to have a substance, appearing like melted lead, poured into their mouth, I have never undertaken to make use of it in my practice; as gold leaf, or fine tinfoil, may be injected so successfully, and be retained very securely for many years. On these accounts, I only noticed it in my work, as the plan recommended and successfully practised by Mr. Pepys; and, therefore, I must observe, that I do not advocate any plan of my own.

The teeth, in which Mr. Pepys has used it, have been those of the lower jaw, in which the caries has been in the centre of the crown, having the sides perfect; and, also, the caries has not been so deep as to have rendered the nerve bare, or so much exposed as to be painfully affected by the temperature; for certainly, although the heat, at which the metal goes into fusion, is only that of boiling water, and will remain fluid, at a less degree of heat, yet, if the nerve of a tooth be very sensible, the excitement likely to proceed from so much heat, would occasion great pain.

Now, in all these cases, the nerve was not in that state; and, I understand, that the sensation on the tooth, was not greater than that which is often felt by incautiously putting some article of food into the mouth, when it has almost immediately been taken out of the pot. Likewise, Mr. P. always defended the mouth, by placing some linen on each side of the tooth, over the gums and the lips, lest any portion of the metal should drop; an accident which, if it did occur, could only scald a little; but it would prevent the success of the operation, by causing the person to draw away the head suddenly.

Having thus defended the mouth, he wipes out the cavity of the tooth, and then introduces a small quantity of powdered rosin, by means of a camel's hair-pencil; then, with a common scoop director, he pours in the metal, until the cavity is perfectly filled: the metal soon becomes solid, and in cooling forms a little button; and in this manner it has remained in some teeth for several years. The addition of the rosin appears to obviate Mr. W's objection, of its not being successful, by its loss of bulk, whilst it passes into the state of congelation.

With respect to Mr. Wawn's improvement, in the claw of the key instrument, I beg leave to observe, that I presume it is a very useful addition to that instrument.

Indeed,

Indeed, I believe it is universally the case, that every *operator of experience* has some improved, or favourite instrument, which he prefers to all others.

I am, &c.

JOSEPH FOX.

*Dover, November* 18, 1807.

